# The effect of vascular risk factors on the efficacy of endolymphatic sac decompression surgery for Meniere’s disease: a retrospective cohort study

**DOI:** 10.3389/fneur.2023.1194456

**Published:** 2023-05-25

**Authors:** Yiling Li, Fengyuan Gong, Yangyang Guo, Xianrong Xu, Cuicui Wang, Zhanguo Jin

**Affiliations:** ^1^Air Force Clinical College, The Fifth School of Clinical Medicine, Anhui Medical University, Hefei, Anhui, China; ^2^Vertigo Clinic/Research Center of Aerospace Medicine, Air Force Medical Center, PLA, Beijing, China; ^3^Graduate School, Hebei North University, Zhangjiakou, China

**Keywords:** Meniere’s disease, endolymphatic sac decompression, vascular risk factor, vertigo, quality of life

## Abstract

**Objectives:**

This study aimed to investigate the effect of vascular risk factors on the outcomes of endolymphatic sac decompression (ESD) surgery in patients with Meniere’s disease.

**Methods:**

The study included 56 patients with Meniere’s disease, who had undergone unilateral ESD surgery. The patients’ vascular risk factors were assessed based on the preoperative 10-year atherosclerotic cardiovascular diseases risk classification. Those with no or low risk were defined as the low-risk group, while those with medium, high, or very high risk were defined as the high-risk group. The correlation between the vascular risk factors and ESD efficacy was evaluated by the comparison of vertigo control grade between the two groups. The functional disability score was also assessed to investigate whether ESD improved the quality of life in Meniere’s disease patients with vascular risk factors.

**Results:**

After ESD, 78.95 and 81.08% of patients from the low-risk and high-risk groups, respectively, demonstrated at least grade B vertigo control; no statistically significant difference was observed (*p* = 0.96). The postoperative functional disability scores in both groups were significantly lower compared with those before surgery (*p* < 0.01), with a median decrease of two (1, 2) points in both groups. No statistically significant difference between the two groups was observed (*p* = 0.65).

**Conclusion:**

Vascular risk factors have little effect on the efficacy of ESD in patients with Meniere’s disease. Patients with one or more vascular risk factors can still experience a not poor vertigo control and improved quality of life after ESD.

## 1. Introduction

Meniere’s disease (MD) is an inner ear disease characterized by paroxysmal vertigo, fluctuating hearing loss, tinnitus, and ear fullness (1). The etiology of MD is unknown, and the main pathological feature is membranous labyrinthine hydrops which increased pressure in the inner ear, bringing vertigo attack, Reissner membrane displacement, and hair cell damage (2). Vertigo control is the main demand of most MD patients while protection of hearing and vestibular function is also expected. For patients with frequent vertigo attacks and ineffective non-surgical treatment for 6 months, surgical treatment can be performed (3).

The surgical methods could be divided into two types according to whether the vestibular function was preserved. Vestibular function destructive surgeries such as semicircular canal occlusion control vertigo by damaging the vestibular sensory organ, while vestibular function protection surgeries such as endolymphatic sac decompression (ESD) do it by reducing the inner pressure. With the latter surgery, the audio-vestibular function is preserved to the maximum extent and the time and money spent on vestibular rehabilitation required due to surgical injury will be avoided. However, the application of ESD is limited by its uncertain curative effect (4). Most of the studies reported a vertigo control rate of approximately 80% (5), and some reported only 66% (6) or even lower (7), suggesting poor surgical efficacy in some patients. Previous studies have shown that patients with negative results on glycerol testing or cochlear electrograms (8) and those with non-ascending audiograms (9) or stage IV hearing (average hearing threshold >70 dB) (3, 10) demonstrate low vertigo control rates after surgery. The characteristics of the population for which this surgery is applicable are still being explored.

Meniere’s disease frequently occurs at the age of 40–60 years (2). Compared with young healthy adults, a larger proportion of patients with MD have one or more vascular risk factors such as hyperlipidemia, hypertension, diabetes, and smoking history (11, 12). Vascular risk factors play an important role in the development of MD. A reduction in the blood supply of the inner ear or blockage of the venous return owing to microvascular injury, oxidative stress, atherosclerotic plaque formation, and micro thrombosis affects the balance in the production and absorption of endolymphatic fluid, thereby increasing the risk of endolymphatic hydrops (13–15). Previous studies have found that patients with vascular risk factors experience vertigo attacks more frequently (16) and demonstrate a poor response to routine treatment of medication (17–19). However, whether patients with vascular risk factors demonstrate poor control after ESD remains unclear. One of the difficulties of conducting research in this area may be the lack of indicators for an overall assessment of vascular risk factors.

Numerous vascular risk factors including hyperlipidemia, hypertension, diabetes, a smoking history, and other factors increase the risk of ischemic cardiovascular disease (20); it is, therefore, inappropriate to consider only a single factor. Fortunately, the 10-year atherosclerotic cardiovascular disease (ASCVD) risk classification was recently developed to provide an overall assessment of vascular risk factors. It is recommended by the Chinese Guidelines for the Prevention of Cardiovascular Diseases (2017) (20). It has been established based on results from long-term cohort studies on cardiovascular disease incidence risk in China; it is, therefore, applicable to the Chinese population and has been widely used in clinical practice. By using it for the overall evaluation of patients and comparing the differences in surgical efficacy among patients with different ASCVD risks, the effect of vascular risk factors on the efficacy of ESD could be figured out.

For MD patients, unpredictable and disabling vertigo episodes can disrupt normal work and life, bringing great pain (21). Therefore, this study aimed to provide a reference for the ESD selection of patients with vascular risk factors in the hope of achieving a balance between function preservation and vertigo control. Individualized treatment plans could be created by the evaluation of preoperative data, and unnecessary destructive surgeries chosen due to fear of failed vertigo control could be avoided.

## 2. Materials and methods

### 2.1. Patients

This study is a retrospective cohort study. The patients who underwent unilateral ESD at the Vertigo Clinic/Research Center of Air Force Medical Center from 2013 to 2020 were included as research subjects.

**Figure 1:**
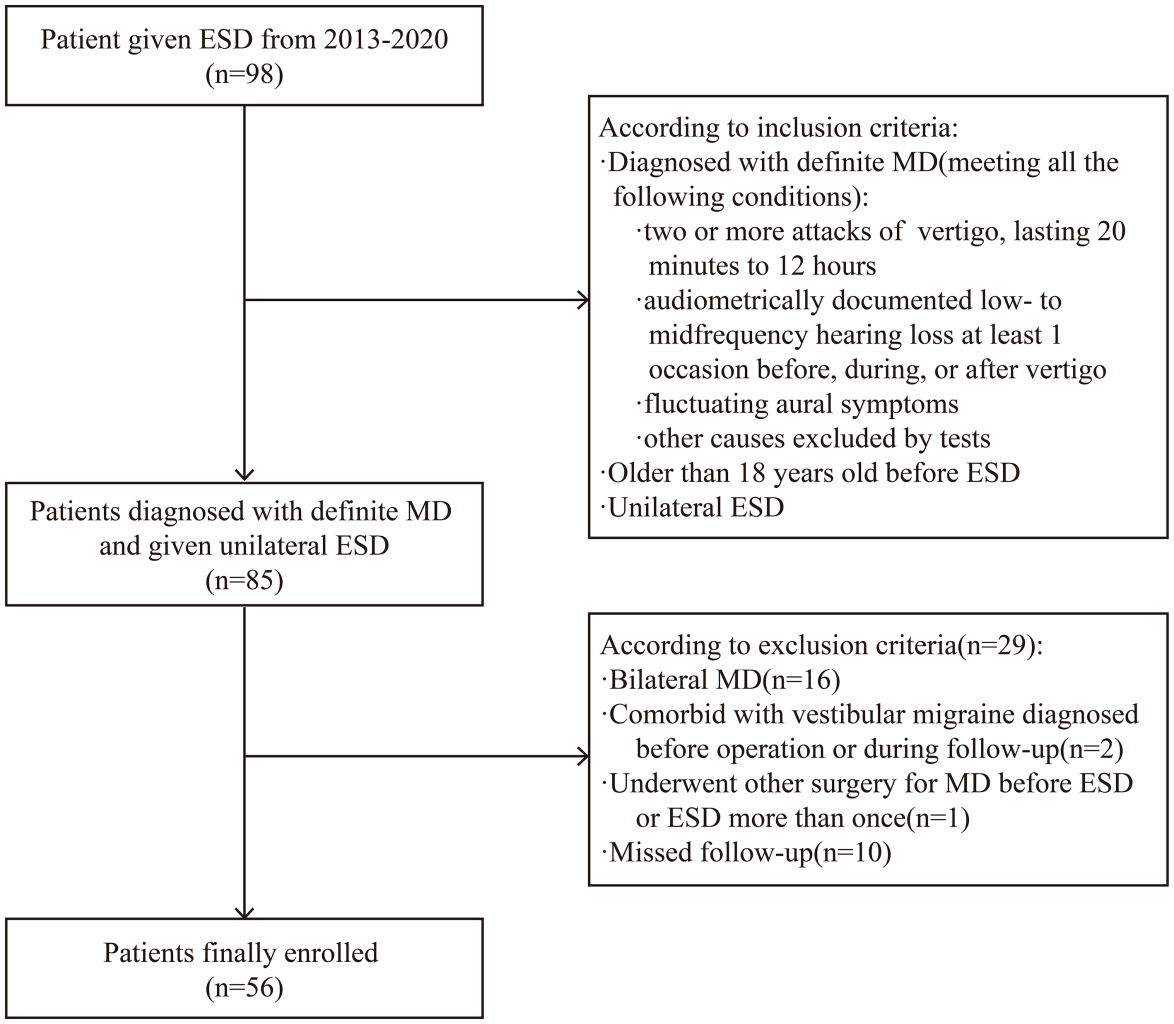
Flowchart of patient inclusion and exclusion. ESD, endolymphatic sac decompression. MD, Meniere’s disease.

As shown in Figure 1, patients conforming to the definite MD diagnosis criteria of the Clinical Practice Guideline: Meniere’s Disease (2020) (4) and with an age at operation exceeding 18 years were included in this study. Patients with bilateral MD, those with comorbid vestibular migraine diagnosed before the operation or during follow-up (22), those who had undergone other surgeries for MD (except intratympanic injection) or ESD more than once, and those who underwent follow-up for less than 2 years or failed to attend follow-up were excluded.

### 2.2. Methods

#### 2.2.1. Surgical procedure and postoperative medications of ESD

ESD was performed based on the method reported by Shambaugh (23) in 1975. A C-shaped incision was placed behind the patient’s ear. The area of the mastoid process extending from the temporal line to the mastoid tip was then ground under the microscope. The mastoid cells were fully cleared, and the mastoid process contoured; this exposed the lateral and posterior semicircular canals and the sigmoid sinus. The endolymphatic sac was found anterior to the sigmoid sinus and between the posterior semicircular canal and the line extending from the lateral semicircular canal. An area of bone measuring 1 × 1 cm^2^ was then removed to fully expose the endolymphatic sac.

Data pertaining to medications used within 14 days after ESD (including betahistine, diuretics, and hormones) were obtained.

#### 2.2.2. The evaluation of ASCVD risk in 10 years

The patients’ ASCVD risk in 10 years was evaluated based on their smoking history, blood lipid test results, sex, age, and previous history of hypertension and diabetes (20) (Table 1). The patients with no and low risk were classified as one cohort (low-risk group), while those having medium, high, and very high risk were classified as another cohort (high-risk group). The effect of ESD was compared between the low-and high-risk groups.

**Table 1:** Atherosclerotic cardiovascular diseases (ASCVD) risk in a 10-year classification.

The number of risk factors		Serum cholesterol (mmol/L)		
		3.1 ≤ TC <4.1 or 1.8 ≤ LDL-C < 2.6	4.1 ≤ TC <5.2 or 2.6 ≤ LDL-C < 3.4	5.2 ≤ TC <7.2 or 3.4 ≤ LDL-C < 4.9
No hypertension	0 ~ 1	Low risk (<5%)	Low risk (<5%)	Low risk (<5%)
	2	Low risk (<5%)	Low risk (<5%)	Medium risk (5% ~ 9%)
	3	Low risk (<5%)	Medium risk (5% ~ 9%)	Medium risk (5% ~ 9%)
Hypertension	0	Low risk (<5%)	Low risk (<5%)	Low risk (<5%)
	1	Low risk (<5%)	Medium risk (5% ~ 9%)	Medium risk (5% ~ 9%)
	2	Medium risk (5% ~ 9%)	High risk (5% ~ 9%)	High risk (5% ~ 9%)
	3	High risk (5% ~ 9%)	High risk (5% ~ 9%)	High risk (5% ~ 9%)

TC, total cholesterol. LDL-C, low-density lipoprotein cholesterol. HDL-C, high-density lipoprotein cholesterol. Risk factors include smoking, low HDL-C, age ≥ 45 (men), and age ≥ 55 (women). Patients with a history of ASCVD were directly classified as very high risk. Patients with any of the following were directly classified as high risk: history of diabetes and age ≥ 40, LDL-C ≥ 4.9 mmol/L or TC ≥ 7.2 mmol/L, systolic pressure ≥ 180 mmHg or diastolic pressure ≥ 110 mmHg, and smoking more than 30 cigarettes a day.

#### 2.2.3. The evaluation of audiometric examination

Pure tone audiometry was performed on all the patients enrolled and the worst result during 6 months before ESD was used to evaluate their hearing. The preoperative hearing threshold was defined as a pure tone average (PTA) of 0.5, 1, and 2 kHz, and the hearing stage was classed based on it (3). Audiograms were divided into ascending and non-ascending types (24). The glycerol test was performed according to our previous research (25).

#### 2.2.4. Subtype classification of MD

The patients with non-classical MD were classified (26) as having MD with migraine (22), autoimmune MD, or familial MD based on their medical and family history. The remainder were classified as having classical MD.

### 2.3. The evaluation of the ESD effect

#### 2.3.1. Vertigo control

Vertigo control was evaluated by comparing the frequency of episodes experienced before and after surgery (27, 28). The value was calculated as follows:

Numerical value = (X/Y) × 100 (rounded to the nearest whole number), where X was the average number of definitive spells per month during 18–24 months after therapy, and Y was the average number of definitive spells per month during 0–6 months before therapy. The vertigo control class was divided into six levels based on numerical value (Supplementary Table S1), and the basic control rate of vertigo was defined as the proportion of patients with grade A and B vertigo control (numerical value ≤ 40).

#### 2.3.2. Dysfunction

The dysfunction score levels (one to six points) using the functional level scale (Supplementary Table S2) and the differences between preoperative and postoperative dysfunction were evaluated based on the functional level scale (27, 28).

### 2.4. Statistical methods

The SPSS 26.0 software package was used for statistical analysis. The *t*-test was used to compare two groups of normal data, and a non-parametric test was used to compare two groups of non-normal or grade data. The chi-square test was used to compare rates between the two groups; *p* < 0.05 was considered statistically significant.

## 3. Results

### 3.1. Basic patient characteristics

A total of 56 patients including 26 (46.43%) men and 30 (53.57%) women fulfilled the inclusion and exclusion criteria (Figure 1). The age at operation was 23–68 (average age: 50.32 ± 11.13) years and the duration of follow-up extended between 2 and 9 years, with a median duration of 6.73 (5.57 and 7.79) years. Complete preoperative and postoperative dysfunction score data were available for 43 patients. The low-and high-risk groups demonstrated statistically significant differences in terms of sex, age at operation, and course of disease (*P* < 0.05; Table 2). These variables were included during multivariate analysis to exclude their influence on the results. There was no statistical difference between the two groups in terms of glycerol test positivity rates, the subtype of MD, preoperative hearing threshold, hearing stage, type of audiogram, preoperative frequency of vertigo, preoperative dysfunction score, and medications used after ESD (*p* > 0.05).

**Table 2:** Comparison of baseline data between the low-and high-risk groups.

	Low-risk group (*N* = 37)	High-risk group (*N* = 19)	*P*
Sex (*N* [%])			0.008
Women	25 (67.6%)	5 (26.3%)	
Men	12 (32.4%)	14 (73.7%)	
Age/Yr (Mean ± SD)	47.59 ± 11.06	55.63 ± 9.44	0.009
Course/Yr (Median [P25, P75])	2.00 (1.00,4.00)	4.00 (2.50,6.50)	0.024
Follow-up/Yr (Median [P25, P75])	6.55 (5.47,7.48)	6.94 (5.80,8.30)	0.416
Subtype of MD (*N* [%])			1.000
Classical MD	30 (81.1%)	16 (84.2%)	
Non-classical MD	7 (18.9%)	3 (15.8%)	
Glycerol test (*N* [%])			
Positive	14 (37.8%)	9 (47.4%)	0.689
Negative	23 (62.2%)	10 (52.6%)	
PTA (Mean ± SD)	50.32 ± 17.62	54.68 ± 14.82	0.360
Hearing stage (*N* [%])			0.378
I	4 (10.8%)	1 (5.3%)	
II	7 (18.9%)	1 (5.3%)	
III	23 (62.2%)	16 (84.2%)	
IV	3 (8.1%)	1 (5.3%)	
Type of audiogram (*N* [%])			0.794
Ascending	9 (24.3%)	6 (31.6%)	
Non-ascending	28 (75.7%)	13 (68.4%)	
Frequency (Median [P25, P75])	6.00 (2.00,12.00)	4.00 (1.50,10.50)	0.855
Dysfunction score (*N* [%])	28	15	0.923
2	9 (32.1%)	4 (26.7%)	
3	14 (50%)	8 (53.3%)	
4	3 (10.7%)	2 (13.3%)	
5	1 (3.6%)	1 (6.7%)	
6	1 (3.6%)	0 (0%)	
Medication (*N* [%])	29 (85.29%)	17 (94.44%)	0.599
Betahistine	23 (67.65%)	17 (94.44%)	0.066
Diuretic	11 (32.35%)	6 (33.33%)	0.943
Hormone	11 (32.35%)	5 (27.78%)	0.734

N, number. Yr, year. SD, standard deviation. Age, age at operation. Course, Course of Meniere’s disease. MD, Meniere’s disease. PTA, pure tone average of 0.5, 1, and 2 kHz. Frequency: the number of monthly vertigo attacks during the 6 months before the operation. Medication: the use of betahistine, diuretic, or hormone after the operation.

### 3.2. Comparison of the effect of ESD between low-and high-risk groups

The basic control rates of vertigo in the low-and high-risk groups were 78.95 and 81.08%, respectively (Table 3); no statistical differences were observed between the vertigo control classes of the two groups (*Z* = –0.07, *p* = 0.96). The risk of failing to achieve basic control of vertigo did not significantly increase in the high-risk group (relative risk = 0.90, 95% confidence interval: 0.30–2.69, *p* = 1.00).

**Table 3:** Comparison of postoperative vertigo control classes between low-and high-risk groups.

Vertigo control class	Low-risk group (*N* [%]) (*N* = 37)	High-risk group (*N* [%]) (*N* = 19)
A	22 (59.5%)	12 (63.2%)
B	8 (21.6%)	3 (15.8%)
C	1 (2.7%)	0 (0.0%)
D	1 (2.7%)	0 (0.0%)
E	0 (0.0%)	0 (0.0%)
F	5 (13.5%)	4 (21.1%)

N, number of patients.

Compared with the scores before surgery, the postoperative dysfunction scores decreased significantly among patients from both groups (low-risk group: *Z* = –4.51, *p* < 0.01; high-risk group: *Z* = –3.11, *p* < 0.01); both groups demonstrated a median decrease of two (1, 2) points and the difference was not statistically significant (*Z* = –0.46, *p* = 0.65).

### 3.3. Multivariate binary logistic regression analysis

Considering the failure of basic vertigo control as a positive event, the multivariate binary logistic regression equation was constructed using the following variables: risk of ASCVD in 10 years, sex, age at operation, and course of the disease. The Hosmer-Lemeshow test showed a good model fit (*p* = 0.51). After adjusting for sex, age at operation, and course of the disease, the results showed that inclusion in the high-risk group had no statistically significant impact on the failure of basic vertigo control (*p* = 0.13; Table 4).

**Table 4:** Multivariate binary logistic regression model for basic vertigo control.

Variables		*b*	SE of *b*	*X^2^*	*P*	OR (95%CI)
Sex						
	Women					
	Men	1.66	0.97	2.93	0.09	5.27 (0.79, 35.33)
Age at operation/Yr		0.10	0.05	5.26	0.02	1.11 (1.02, 1.21)
The course of disease/Yr		0.05	0.04	1.61	0.20	1.05 (0.97, 1.14)
Risk of ASCVD in 10 years						
	Low risk					
	High risk	−1.62	1.08	2.24	0.13	0.20 (0.02, 1.65)

SE, standard error. OR, odds ratio. CI, confidence interval.

## 4. Discussion

In this retrospective cohort study, the results of statistical analyses showed that the basic vertigo control rate was nearly 80% in both low-and high-risk groups. There was no statistical difference between the two groups regardless of whether variables such as sex, age at operation, and course of disease were controlled. In addition, the postoperative dysfunction score decreased significantly in both groups. This suggested that the existence of vascular risk factors did not worsen surgical efficacy in these patients. The patients experienced good vertigo control and the disease-related dysfunction had been resolved.

Since the introduction of ESD in 1927, surgical outcomes have varied across different studies (5–7). As this may be attributed to differences in the included patient groups, it is essential to identify the clinical characteristics of patients who are appropriate candidates for this operation; this may help predict the surgical benefit and reduce unnecessary surgical intervention (29). The age associated with a high incidence of MD partly overlaps with that associated with a high incidence of vascular risk factors (such as hypertension, hyperlipidemia, and diabetes). In this context, patients with vascular risk factors have more severe symptoms and a poorer prognosis. As vascular risk factors may affect surgical efficacy, this study compared the surgical efficacy between low-and high-risk patients and found vascular risk factors to have no significant effect.

This finding, which was inconsistent with our expectations, demonstrated the complexity of the mechanisms involved in the etiopathogenesis of MD. Findings from existing studies suggest that patients with a greater number of vascular risk factors obtain less benefit from routine treatment; however, the patients in this study experienced good outcomes after ESD. This may be attributed to the fact that the operation alleviates the rapid expansion of membranous labyrinthine hydrops rather than cures it. Notably, the membranous labyrinthine hydrops caused by vascular risk factors or other causes persists after surgery (30). The operation just prevents further aggravation of the condition owing to arteriovenous mixing, which may result from endolymphatic sac vessel reflux to the inner ear due to excessive pressure (31). Current research suggests that vertigo attacks are related to the rupture of the membranous labyrinth which is caused by an acute increase in endolymph rather than hydrops itself (32), which happened in 12.5–30.0% of healthy individuals (33). The operation provides space for the expansion of the endolymph sac; this in turn prevents any serious organ damage caused by rapid hydrops and thereby achieves the goal of reducing vertigo attacks. Patients with one or more vascular risk factors may therefore still obtain good control of vertigo despite the existence of membranous labyrinthine hydrops.

The greatest strength of our study is the control of confounding bias. As numerous factors may influence the efficacy of ESD, it is difficult to identify the actual impact of vascular risk factors. Therefore, the baseline data from the low-and high-risk groups were compared to control interference from heterogeneity in demographic characteristics (sex, age, and time of follow-up), etiology (a subtype of MD), state of hydrops (results of the glycerol test), progress and severity of MD (course of disease, hearing stage, type of audiogram, preoperative hearing threshold, preoperative frequency of vertigo, and preoperative dysfunction score), and medications used after ESD. For variables that demonstrated an imbalance in distribution between the groups (such as sex, age, and course of the disease), multifactorial analysis was performed to control their influence. Compared with single factor analysis, which does not consider the impact of other factors on ESD, our analysis fully considered various factors that affect postoperative vertigo control (8–10, 34) and identified the exclusive impact of vascular risk factors with considerable reliability.

Our study had certain limitations. First, the sample size was relatively small; the findings, therefore, need to be validated in large-scale and multi-center studies. Second, the impact of vasopressin on the ESD effect was not controlled because of the lack of prospective design. Vasopressin was proved to correlate with MD attack by overexpressing vasopressin type-2 receptor and subsequently activating aquaporin-2 (35, 36), which may interfere with the evaluation of ESD efficacy. Third, on ASCVD risk stratification, only one patient in this study demonstrated no risk in 10 years; the sample size for comparison and analysis of surgical effects in patients with/without vascular risk factors was therefore limited. Fourth, the control of vascular risk factors during follow-up was not assessed in this study, which limited further demonstration of the vascular risk factors’ impact on ESD efficacy. A prospective study is needed to confirm if the changes in vascular risk factors can lead to changes in ESD efficacy. Last but not least, ASCVD risk in 10 years may not be an optimal indicator for a comprehensive evaluation of vascular risk factors. It focuses on the occurrence of ASCVD, in which the vascular lesions are more severe than those of MD. Further research will therefore be performed using more appropriate indicators.

Finally, it is worth noting that maintaining a healthy lifestyle and control of vascular risk factors are still necessary for MD patients because of the high disability rate and mortality of cerebral or myocardial infarction even though the finding above suggests that ESD efficacy is not much affected by vascular risk factors.

## 5. Conclusion

In conclusion, ESD may effectively reduce vertigo attacks and dysfunction in patients with MD, and its efficacy is not reduced by an increase in vascular risk factors. The findings from this study may increase confidence regarding the use of ESD in patients with MD, thereby preventing unnecessary destructive surgeries.

## Data availability statement

The raw data supporting the conclusions of this article will be made available by the authors, without undue reservation.

## Ethics statement

The studies involving human participants were reviewed and approved by Medical Ethics Committee of Air Force Medical Center. Written informed consent for participation was not required for this study in accordance with the national legislation and the institutional requirements.

## Author contributions

ZJ and CW designed the research. FG, YG, and YL prepared the material, collected the data, and analyzed the data. YL prepared the figures. XX and YL wrote the first draft of the manuscript. All authors contributed to the study’s conception and design, commented on previous versions of the manuscript, and read and approved the final manuscript.

## Funding

This study has been funded by the major program of the PLA logistics department (number: BKJ19J020).

## Conflict of interest

The authors declare that the research was conducted in the absence of any commercial or financial relationships that could be construed as a potential conflict of interest.

## Publisher’s note

All claims expressed in this article are solely those of the authors and do not necessarily represent those of their affiliated organizations, or those of the publisher, the editors and the reviewers. Any product that may be evaluated in this article, or claim that may be made by its manufacturer, is not guaranteed or endorsed by the publisher.
