# Supplementary material for: The effect of vascular risk factors on the efficacy of endolymphatic sac decompression surgery for Meniere’s disease: a retrospective cohort study
Source: Front Neurol. 2023 May 25;14:1194456. doi: 10.3389/fneur.2023.1194456 (PMC10248514; doi:10.3389/fneur.2023.1194456)
Supplement: Supplementary file 1 [file Table_1.docx]

Supplementary Material

Effect of Vascular Risk Factors on Efficacy of Endolymphatic Sac Decompression Surgery for Meniere’s Disease: A Retrospective Cohort Study

Li Yiling, Gong Fengyuan, Xu Xianrong, Wang Cuicui*, Jin Zhanguo*

*** Correspondence:** Jin Zhanguo：[ccjzg@126.com](mailto:ccjzg@126.com)； Wang Cuicui：cuicuiwang_169@163.com

**Supplementary Table 1.** Vertigo control classes.

| Numerical value | Class |
| --- | --- |
| 0 | A |
| 1-40 | B |
| 41-80 | C |
| 81-120 | D |
| ＞120 | E |
| Secondary treatment initiated due to disability from vertigo | F |

Numerical value = (X/Y) × 100 (rounded to the nearest whole number), where X was the average number of definitive spells per month during 18-24 months after therapy and Y was the average number of definitive spells per month during 0-6 months before therapy.
