# Supplementary material for: The effect of vascular risk factors on the efficacy of endolymphatic sac decompression surgery for Meniere’s disease: a retrospective cohort study
Source: Front Neurol. 2023 May 25;14:1194456. doi: 10.3389/fneur.2023.1194456 (PMC10248514; doi:10.3389/fneur.2023.1194456)
Supplement: Supplementary file 2 [file Table_2.docx]

Supplementary Material

Effect of Vascular Risk Factors on Efficacy of Endolymphatic Sac Decompression Surgery for Meniere’s Disease: A Retrospective Cohort Study

Li Yiling, Gong Fengyuan, Xu Xianrong, Wang Cuicui*, Jin Zhanguo*

*** Correspondence:** Jin Zhanguo：[ccjzg@126.com](mailto:ccjzg@126.com)； Wang Cuicui：cuicuiwang_169@163.com

**Supplementary Table 2.** Functional level scale.

| Score | Regarding my current state of overall function, not just during attacks (check the one that best applies): |
| --- | --- |
| 1 | My dizziness has no effect on my activities at all. |
| 2 | When I am dizzy I have to stop what I am doing for a while, but it soon passes and I can resume activities. I continue to work, drive, and engage in any activity I choose without restriction. I have not changed any plans or activities to accommodate my dizziness. |
| 3 | When I am dizzy I have to stop what I am doing for a while, but it does pass and I can resume activities. I continue to work, drive, and engage in most activities I choose, but I have had to change some plans and make some allowance for my dizziness. |
| 4 | I am able to work, drive, travel, take care of a family, or engage in most essential activities, but I must exert a great deal of effort to do so. I must constantly make adjustments in my activities and budget my energies. I am barely making it. |
| 5 | I am unable to work, drive, or take care of a family. I am unable to do most of the active things that I used to. Even essential activities must be limited. I am disabled. |
| 6 | I have been disabled for 1 year or longer and/or I receive compensation (money) because of my dizziness or balance problem. |
